# IL-17/IL-10 double-producing T cells: new link between infections, immunosuppression and acute myeloid leukemia

**DOI:** 10.1186/s12967-015-0590-1

**Published:** 2015-07-15

**Authors:** Gerardo Musuraca, Serena De Matteis, Roberta Napolitano, Cristina Papayannidis, Viviana Guadagnuolo, Francesco Fabbri, Delia Cangini, Michela Ceccolini, Maria Benedetta Giannini, Alessandro Lucchesi, Sonia Ronconi, Paolo Mariotti, Paolo Savini, Monica Tani, Pier Paolo Fattori, Massimo Guidoboni, Giovanni Martinelli, Wainer Zoli, Dino Amadori, Silvia Carloni

**Affiliations:** Hematology Unit, Istituto Scientifico Romagnolo per lo Studio e la Cura dei Tumori (IRST) IRCCS, Meldola, Italy; Biosciences Laboratory, Istituto Scientifico Romagnolo per lo Studio e la Cura dei Tumori (IRST) IRCCS, Meldola, Italy; Department of Hematology and Oncological Sciences ‘L. and A. Seràgnoli’, University of Bologna, Bologna, Italy; Internal Medicine Unit, Infermi Hospital, Faenza, Italy; Haematology Unit, Santa Maria delle Croci Hospital, Ravenna, Italy; Immunotherapy Unit, Istituto Scientifico Romagnolo per lo Studio e la Cura dei Tumori (IRST) IRCCS, Meldola, Italy; Department of Medical Oncology, Istituto Scientifico Romagnolo per lo Studio e la Cura dei Tumori (IRST) IRCCS, Meldola, Italy

**Keywords:** AML, Infections, IL17+/IL10+ T cells, Immunosuppression, Leukemia immunoescape

## Abstract

**Background:**

Acute myeloid leukemia (AML) is an incurable disease with fatal infections or relapse being the main causes of death in most cases. In particular, the severe infections occurring in these patients before or during any treatment suggest an intrinsic alteration of the immune system. In this respect, IL-17-producing T helper (Th17) besides playing a key role in regulating inflammatory response, tumor growth and autoimmune diseases, have been shown to protect against bacterial and fungal pathogens. However, the role of Th17 cells in AML has not yet been clarified.

**Methods:**

T cell frequencies were assessed by flow cytometry in the peripheral blood of 30 newly diagnosed AML patients and 30 age-matched healthy volunteers. Cytokine production was determined before and after culture of T cells with either *Candida Albicans* or AML blasts. Statistical analyses were carried out using the paired and unpaired two-tailed Student’s t tests and confirmed with the non parametric Wilcoxon signed-rank test.

**Results:**

A strong increase of Th17 cells producing immunosuppressive IL-10 was observed in AML patients compared with healthy donors. In addition, stimulation of AML-derived T cells with a *Candida albicans* antigen induced significantly lower IFN-γ production than that observed in healthy donors; intriguingly, depletion of patient Th17 cells restored IFN-γ production after stimulation. To address the role of AML blasts in inducing Th17 alterations, CD4+ cells from healthy donors were co-cultured with CD33+ blasts: data obtained showed that AML blasts induce in healthy donors levels of IL-10-producing Th17 cells similar to those observed in patients.

**Conclusions:**

In AML patients altered Th17 cells actively cause an immunosuppressive state that may promote infections and probably tumor escape. Th17 cells could thus represent a new target to improve AML immunotherapy.

## Background

Acute myeloid leukemia (AML) is the most common form of acute leukemia in adults [1] and current treatments remain unsatisfactory. Serious infections, resistance to therapy and relapses are the main causes of mortality among patients [2–4]. In particular, the high frequency and severity of infections (especially fungal) before or during chemotherapy are probably due to a severe adaptive immunity dysfunction directly induced by the disease [5, 6]. This immunosuppressive state may also be responsible for the continuous recurrence of AML and for the failure of immunotherapies [7], with the exception of allogeneic transplantation [8].

Although several studies identified different immunosuppressive mechanisms operating in AML [9–13], the precise link between immune alterations, leukemia immune escape and infections has not yet been elucidated. Investigating in this direction, T helper cells seem to be important players with Th17 cells being one of the most intriguing and not fully understood subset so far [14].

Th17 cells show a pleiotropic role in the inflammatory response, autoimmune disorders and tumors [15–19]. In the latter context, they showed remarkable epigenetic plasticity [14] and the ability to transdifferentiate into T helper 1-like cells (secreting IFN-γ and showing tumor suppressor activity) [19–21] or T regulatory (Treg)-like cells (secreting IL-10, with immunosuppressive functions and probably tumor promoter activity) [22–25].

Moreover, Th17 cells have the important task of coordinating the immune defense against bacterial and fungal infections and physiologically protect humans against these diseases [26–28]. However, the behavior of Th17 in several tumor types and hematologic malignancies remains to be clarified [22, 29–31]. In particular, different studies on AML have attributed a controversial pathogenetic role and divergent prognostic values to these cells [31–36] but have not succeeded in establishing a link between the reported alterations and the infections to which these patients are subject.

We showed that Th17 cells with a double production of IL-17 and IL-10 were strongly increased in AML patients and that ex vivo patient immune response to an infectious antigen, such as *Candida Albicans* (*C. Albicans*), was significantly reduced by Th17. Finally, we found that blasts, co-cultured with CD4+ cells from healthy donors, were able to change the frequency and cytokine profile of T cells, and in particular of Th17, in a similar manner to that observed in patients.

All of the above data support the hypothesis that the increase in IL-10+ Th17 cells in AML is a mechanism developed by the disease to create an immunosuppression state which, given the stem-like features and long life of Th17 cells [14, 37], may be durable and ultimately favor infections, protecting leukemia cells from immune control.

## Methods

### Blood samples and PBMCs collection

After obtaining the patient’s informed consent and the approval of the local ethics committee, in accordance with the Declaration of Helsinki, samples of peripheral blood (15–20 ml) were collected from 30 newly diagnosed AML patients before any treatment was started and from 30 age-matched (±10 years) healthy volunteers (HV). AML patients were diagnosed according to the French American-British (FAB) classification system [38]. Patient and HV characteristics are reported in Table 1. Blood samples were collected in sterile EDTA tubes and mononuclear cells (PBMCs) were separated by density gradient centrifugation using Lymphosep (Biowest) and frozen in 90% heat inactivated fetal bovine serum (FBS) (PAA) and 10% dimethylsulfoxide (Sigma Aldrich).

**Table 1:** Patient and HV characteristics

	HV	AML
No. patients	30	30
Gender		
Male	15 (50%)	14 (47%)
Female	15 (50%)	16 (53%)
Median age, years (range)	63 (38–87)	68 (35–85)
Subtype according to FAB classification		
M0–M1		6 (20%)
M2		9 (30%)
M4		4 (13.3%)
M5		9 (30%)
M6		1 (3.3%)
M7		1 (3.3%)
Karyotype		
Normal		14 (47%)
Undefined		4 (13%)
Complex		3 (10%)
Trisomy chr 8		3 (10%)
t(8–21)		2 (7%)
Tetrasomy chr 21		1 (3%)
Inv chr 3		1 (3%)
Del chr 7		1 (3%)
Del chr 20		1 (3%)
Molecular mutations		
FLT3/NPM wt		17 (57%)
FLT3 ITD		4 (13%)
FLT3 mut NPM mut		3 (10%)
FLT3 mut NPM wt		1 (3%)
FLT3 wt NPM mut		1 (3%)
AML-ETO		2 (7%)
Undefined		2 (7%)

*FAB* French-American-British, *chr* chromosome, *t* translocation, *Inv* inversion, *Del* deletion, *wt* wild type, *mut* mutated.

### CD4+ cell isolation and culture

In order to avoid contamination by CD4+ cells that release IL-17, such as macrophages [37], PBMCs were thawed and human CD4+ T cells were isolated by negative depletion of CD8+, CD14+, CD15+, CD16+, CD19+, CD36+, CD56+, CD123+, TCR y/δ and CD235a+, using the CD4+ T cell isolation kit (Miltenyi Biotec). In this way also AML blasts, where present, were included in the subsequent analysis. Cells were cultured in RPMI 1640 medium (PAA) supplemented with 10% heat inactivated FBS, 2 mM l-glutamine (Euroclone), penicillin (100 U/ml) and streptomycin (100 μg/ml) (PAA). CD4+ cells were primed for 24 h at 37°C with IL-6 (30 ng/ml) (Miltenyi Biotec) or TGF-β (10 ng/ml) (Abcam) or a combination of IL-6 and TGF-β. T cells were then incubated for 5 h at 37°C with phorbol 12-myristate-13-acetate (PMA, 50 ng/ml) and ionomycin (1 μg/ml) (Invitrogen) in the presence of GolgiStop Protein Transport Inhibitor (BD Pharmingen). An unstimulated control prepared by incubating CD4+ cells with GolgiStop Protein Transport Inhibitor only was included for each experiment.

### Immunophenotypic analysis of T cells

After stimulation, cells were fixed and permeabilized with Cytofix/Cytoperm (BD Biosciences) then immunophenotyped for intracellular IFN-γ, IL-4 and IL-17A expression using the human TH1/TH2/TH17 phenotyping kit (BD Pharmingen) following the manufacturer’s protocol. For Treg analysis, naïve PBMCs were stained with anti-human FITC CD4 (0.6 μg/ml, clone SK3; BD Biosciences) and anti-human APC-Cy7 CD25 (2.5 μg/ml, clone M-A251; BD Biosciences) for 10 min at 4°C in the dark. After incubation, cells were fixed and permeabilized and then stained with anti-human APC FoxP3 (1:11, clone 3G3; Miltenyi Biotec) for 30 min at 4°C in the dark. Appropriate isotype controls were included for each sample.

### Cytokine secretion analysis

Stimulated CD4+ cells were washed with cold PBS containing 0.5% (v/v) bovine serum albumin (BSA) (Sigma Aldrich) and 2 mM of EDTA and analyzed using human IL-17 and IL-10 secretion assay—detection kits (Miltenyi Biotec). Briefly, cells were stained with IL-17 and IL-10 catch reagents for 5 min on ice, incubated for 45 min at 37°C to allow cytokine secretion and then with anti-human PE IL-17A, anti-human APC IL-10 and anti-human FITC CD4 for 10 min on ice, according to the manufacturer’s instructions. Samples were washed and suspended for flow cytometric analysis.

### CD33+ cells isolation

Circulating CD33+ cells were magnetically isolated from AML PBMCs in two steps: first, CD4+ and blast cells were negatively purified using the T cell isolation kit, as already described; subsequently, CD33+ cells were purified with CD33 MicroBeads kit (Miltenyi Biotec) following the manufacturer’s instructions.

### Direct and indirect allogeneic co-cultures

For direct co-cultures, CD33+ cells isolated from 15 AML patients and allogeneic CD4+ T cells obtained from 15 HV as previously reported were co-seeded in 1:1, 1:5 and 1:10 ratios. For indirect co-cultures, purified CD4+ cells were seeded in the bottom part of the 6-well plates of transwell cell culture system (pore size 0.4 μm; Costar Corp.), whereas CD33+ cells were seeded in the corresponding transwell cell culture inserts. In addition, each cell type was seeded individually in 6-well plates for single culture as control. All samples were cultured in complete medium and stimulated as previously described. At the end of stimulation, T cell immunophenotypic and cytokine secretion analysis was performed.

### T cell activation with *C. Albicans* and isolation of IL-17-secreting cells

CD4+ cells (2.5 × 10^6^) were stimulated for 24 h at 37°C with 1 μg/ml of *C. Albicans* peptides (JPT, Berlin, Germany). During the last 5 h of incubation, cells were maintained in the presence of GolgiStop Protein Transport Inhibitor (BD Pharmingen). Samples were centrifuged at 4°C, incubated with 2 mM of EDTA in PBS for 10 min at 37°C, washed with 0.5% BSA and 0.1% sodium azide in PBS. Cells were then depleted of IL-17-secreting cells using the IL-17 Secretion Assay—Cell Enrichment and Detection Kit (Miltenyi Biotec). The IL-17 specific catch reagent was attached to the cell surface as previously described, after which cells were labeled with anti-human PE IL-17A and stained with anti-PE microbeads. IL-17-secreting cells were separated through two consecutive column runs, according to the manufacturer’s instructions. Negative fraction was cultured for a further 24 h in complete medium supplemented with 1 μg/ml of *C. Albicans* peptides and then analyzed for intracellular IFN-γ expression using the human TH1/TH2/TH17 phenotyping kit (BD Pharmingen). A sample stimulated with *C. Albicans* for 48 h without depletion of IL-17-secreting cells was added as control.

### Flow cytometry

Flow cytometric analysis were performed using a FACSCanto flow cytometer (Becton–Dickinson) equipped with 488 nm (blue) and 633 (red) lasers and 50,000 events were recorded for each sample. The acquisition and analysis gates were set on lymphocytes based on forward (FSC) and side scatter (SSC) properties of cells. FSC and SSC were set in a linear scale. For more extensive analysis, gates were set on CD4+ T cell subsets. Flow cytometry data were analyzed with Diva Software (Becton–Dickinson).

### Statistical analysis

Data were summarized by descriptive statistics (mean ± standard deviation for continued variables and frequency and percentage for categorical variables). Statistical analyses were carried out using the paired and unpaired two-tailed Student’s t tests and confirmed with the non parametric Wilcoxon signed-rank test. *P* values <0.05 were considered as significant.

## Results

### Alterations in the T cells frequency in peripheral blood of AML patients

We first focused on the frequency of CD4+ T cells (Th1, Th2, Th17 and Tregs) in the peripheral blood of 30 newly diagnosed untreated AML patients characterized by karyotype and molecular biology mutations (Table 1) and 30 sex- and age-matched HV. Given the controversies over the different Th17 polarization methods [39–43], we stimulated CD4+ cells isolated through a negative immunomagnetic system in serum-containing media with IL-6 and TGF-β alone and in combination. As no significant differences were observed (data not shown), Th1, Th2 and Th17 analyses were performed with IL-6 alone, thereby reducing the risk of the TGF-β-mediated induction or inhibition of other cytokines.

**Figure 1:**
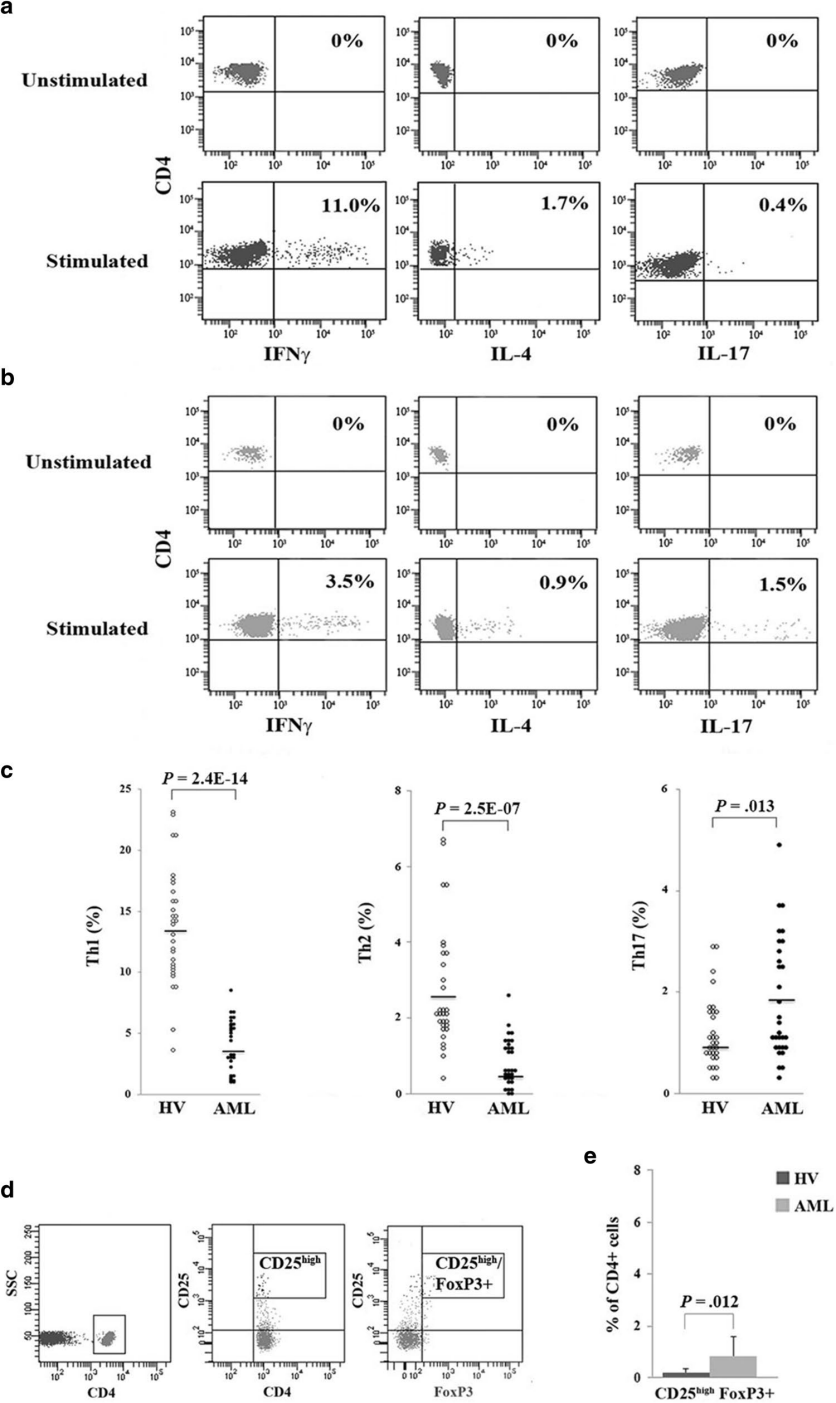
Alterations in T helper cells from HV and untreated AML patients. **a** Representative dot plots of cytokine production by CD4+ cells isolated from a HV before and after in vitro priming with IL-6 and then with phorbol 12-myristate 13-acetate (PMA) and ionomycin (I). **b** Representative dot plots of CD4+ cells from an AML patient before and after stimulation with IL-6 and PMA + I.** c** Pooled data obtained after stimulation from 30 HV (*white circles*) and 30 AML patients (*black circles*) and mean values (*bars*). **d** Gating strategy used to identify the CD4+ CD25^high^FoxP3+ cells. **e** Mean and standard deviation of Treg frequency from HV and AML patients.

As shown in Figure 1a, b, flow cytometric analysis revealed that the frequencies of T helper populations were altered in AML patients in comparison to healthy donors. In particular, Th1 and Th2 percentages were lower in patients (4.0 ± 2.1 and 0.8 ± 0.6% respectively) than in controls (12.8 ± 4.3 and 2.9 ± 1.8% respectively), whereas Th17 cells showed a 1.5-fold increase in AML patients compared to HV (*P* = 0.013) (Figure 1c). All the observed differences were statistically significant.

In addition the frequency of circulating Tregs was performed in unstimulated PBMCs by flow cytometry. CD4+ cells with mean fluorescence intensity of CD25 expression ≥10-fold the negative cut-off were classified as CD25^high^ as previously reported [11] (Figure 1d). Our data highlighted a significantly higher frequency of CD4+ CD25^high^ FoxP3+ cells in AML samples (0.83 ± 0.75%) than in controls (0.19 ± 0.15%) (*P* = 0.012) (Figure 1e), whereas no difference were observed in the frequency of CD4+ CD25+ FoxP3+ cells (P = 0.38, data not shown).

Altered frequency and cytokine profile were already been described in AML but not for all of these subsets of CD4+ cells in the same time and anyway, may suggest a global alteration of patient immune response.

### Increased IL-17/IL-10-secreting cells in the peripheral blood of AML patients

**Figure 2:**
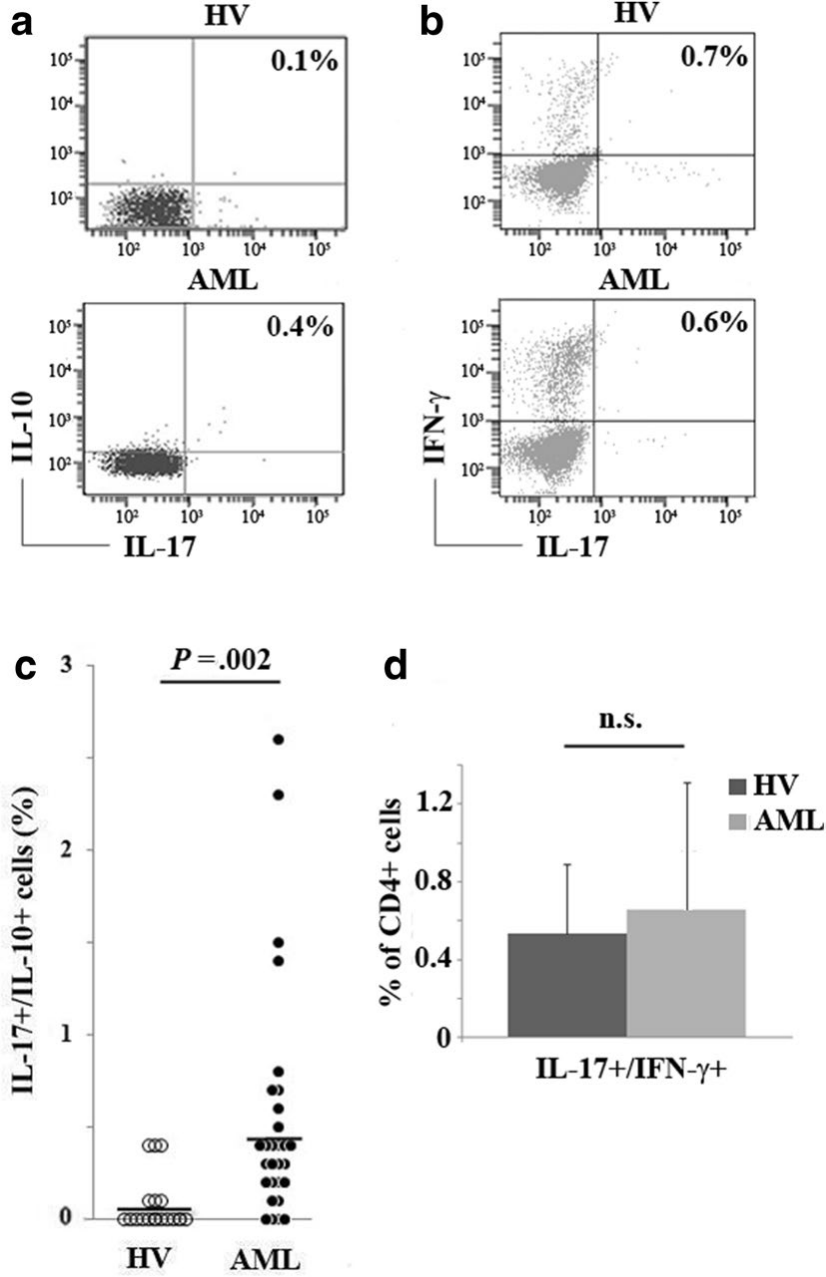
IL-10 and IFN-γ intracellular production by Th17 cells in HV and AML patients. **a** Representative flow cytometric data of IL-17 and IL-10 double release by CD4+ T cells from HV and AML primed with IL-6 and PMA + I. **b** Representative dot plots of IL-17 and IFN-γ simultaneous production from a HV and an AML patient. **c** IL-17/IL-10 pooled data from HV (*white circles*) and AML patients (*black circles*) and mean values (*bars*). The frequency of CD4+ T cells that simultaneously secreted IL-17 and IL-10 was 7.5-fold increased in AML patients compared with HV. **d** IL-17 and IFN-γ production data derived from the entire series are shown as mean and standard deviation The concomitant intracellular production of IFN-γ and IL-17 by CD4+ cells was not significantly different from that of HV or patients (*P* > 0.05).

To deepen this aspect, we also evaluate the ability of the increased Th17 cells to simultaneously produce or secrete other cytokines, focusing on IFN-γ and IL-10 [21, 23–25] (Figure 2a, b). Intriguingly, we observed a strong, statistically significant increase in the frequency of CD4+ IL-17/IL-10 double secreting cells in AML patients compared to HV (0.54 ± 0.63 and 0.075 ± 0.145% respectively; *P* = 0.002) (Figure 2c), whereas the percentage of IL-17 +/IFN-γ+ cells remained unchanged (*P* = 0.39) (Figure 2d). No correlation was observed with specific karyotype, molecular biology mutations or Treg frequency.

The increase of IL-10+ Th17, together with the aforementioned global CD4+ altered frequency, taking into account the singular infectious susceptibility of AML patients and the physiological protective role of Th17 cells, seem to strengthen the hypothesis of a reduced immune response in these patients.

### T cell immune response abnormalities after stimulation with *C. Albicans*

**Figure 3:**
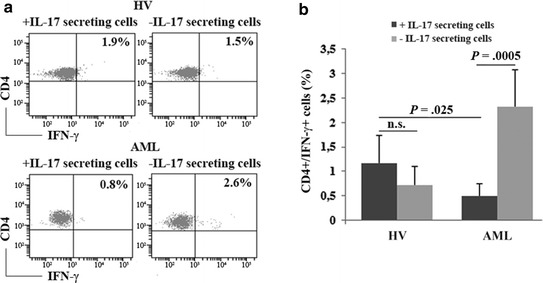
IFN-γ production by CD4+ cells stimulated with *C. Albicans* before and after depletion of IL-17-secreting cells. **a** CD4+ cells were stimulated for 24 h with peptides from *C. Albicans*, then IL-17- secreting cells were then immunomagnetically depleted and negative fraction was cultured for a further 24 h in the presence of *C. Albicans* peptides and analyzed for intracellular IFN-γ expression. Representative dot plots of CD4+/IFN-γ+ cells from HV and AML patient before and after depletion of IL-17-secreting cells are shown. **b** Pooled data from 5 HV and 5 AML patients are shown as mean values and standard deviation (*n.s.* not significant).

In order to evaluate the functional effects of the observed alterations, we analyzed intracellular IFN-γ expression of CD4+ cells from HV and AML patients stimulated with *C. Albicans* with or without depletion of IL-17-secreting cells (Figure 3a).

As shown in Figure 3b, the number of IFN-γ+ cells in not depleted control samples was 2.4-fold lower in AML compared to HV (0.5 ± 0.2 and 1.2 ± 0.6% respectively; *P* = 0.025). Interestingly, in patients the IFN-γ production increased after IL-17-secreting cells depletion (2.3 ± 0.7%; *P* = 0.0005) (Figure 3b). Conversely, in HV, the depletion of IL-17-secreting cells did not induce significant changes in the production of IFN-γ (*P* = 0.21).

This in vitro functional proof is in line with the hypothesis of a reduced immune response in AML caused by altered Th17 cells; on the contrary, in HV the depletion of Th17 cells did not change the IFN-γ response, probably because under physiological conditions, they are not able to make that, alone.

### The impairment of T cells after direct and indirect allogeneic co-culture with CD33+ leukemic blasts

**Figure 4:**
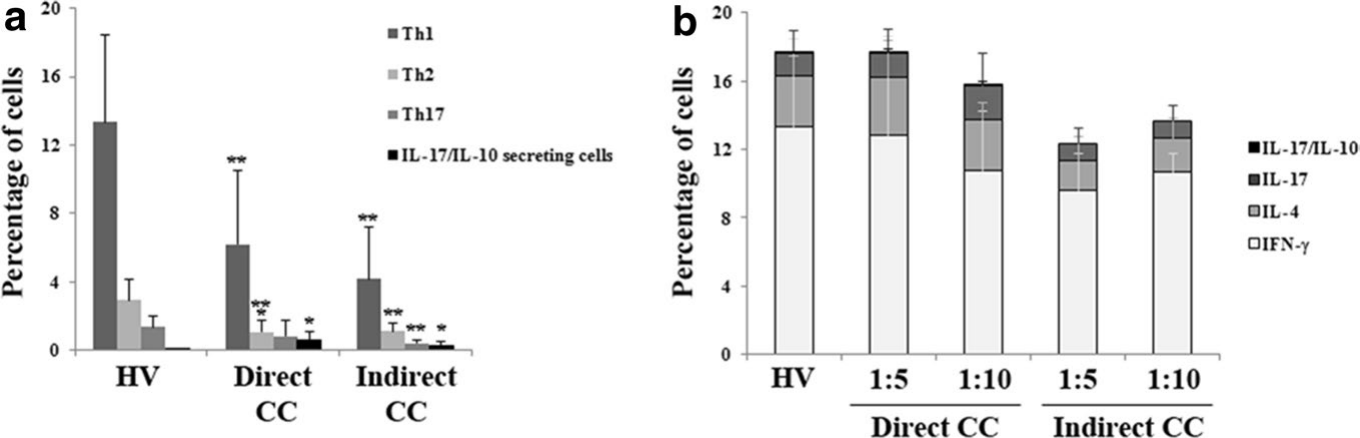
CD33+ blast involvement in inducing alterations in cytokine production by Th1,Th2 and Th17. **a** CD4+ cells isolated from 15 HV and CD33+ cells from 15 AML were co-seeded in a 1:1 ratio in the same well [direct co-culture (CC)] or in a transwell cell culture system (indirect CC). All samples were stimulated with IL-6 and PMA + I as previously described. At the end of stimulation, T cell immunophenotypic and cytokine secretion analysis were achieved. Data (mean and standard deviations) from direct or indirect CC were compared with pooled data obtained from 15 HV (*P < 0.05, **P < 0.005, ***P < 0.0005). **b** HV CD4+ cells and CD33+ blasts were directly or indirectly co-seeded in 1:5 and 1:10 ratios, respectively. All samples were stimulated with IL-6 and PMA + I and then analyzed for IFN-γ, IL-4 and IL-17 production and IL-17 and IL-10 simultaneous secretion. Data are expressed as mean and standard deviation (*P* > 0.05).

To investigate the role of leukemic cells in T cell changes, we performed direct and indirect co-cultures of CD4+ cells obtained from 15 HV and CD33+ blast cells magnetically isolated from 15 AML patients. A cytokine pattern similar to that found in AML patients was observed in CD4+ co-cultured directly at a 1:1 ratio, with a significant reduction in IFN-γ (*P* = 0.0008) and IL-4-positive cells (*P* = 3.2E−05) and a strong increase in the percentage of CD4+ IL-17A/IL-10-secreting cells (0.02 ± 0.05 and 0.56 ± 0.5% before and after co-culture respectively; *P* = 0.008). The above described cytokine alterations were also present when blasts and CD4+ cells were physically separated by a membrane (Figure 4a). In both co-culture methods, these alterations were not observed at CD33+ and CD4+ ratios of 1:5 and 1:10 (Figure 4b).

These finds suggest that the changes observed in CD4+ cells cytokine profile were directly induced by leukemic cells probably by soluble factors.

### Altered T cell cytokine production after depletion of CD33+ blasts in samples from AML patients

**Figure 5:**
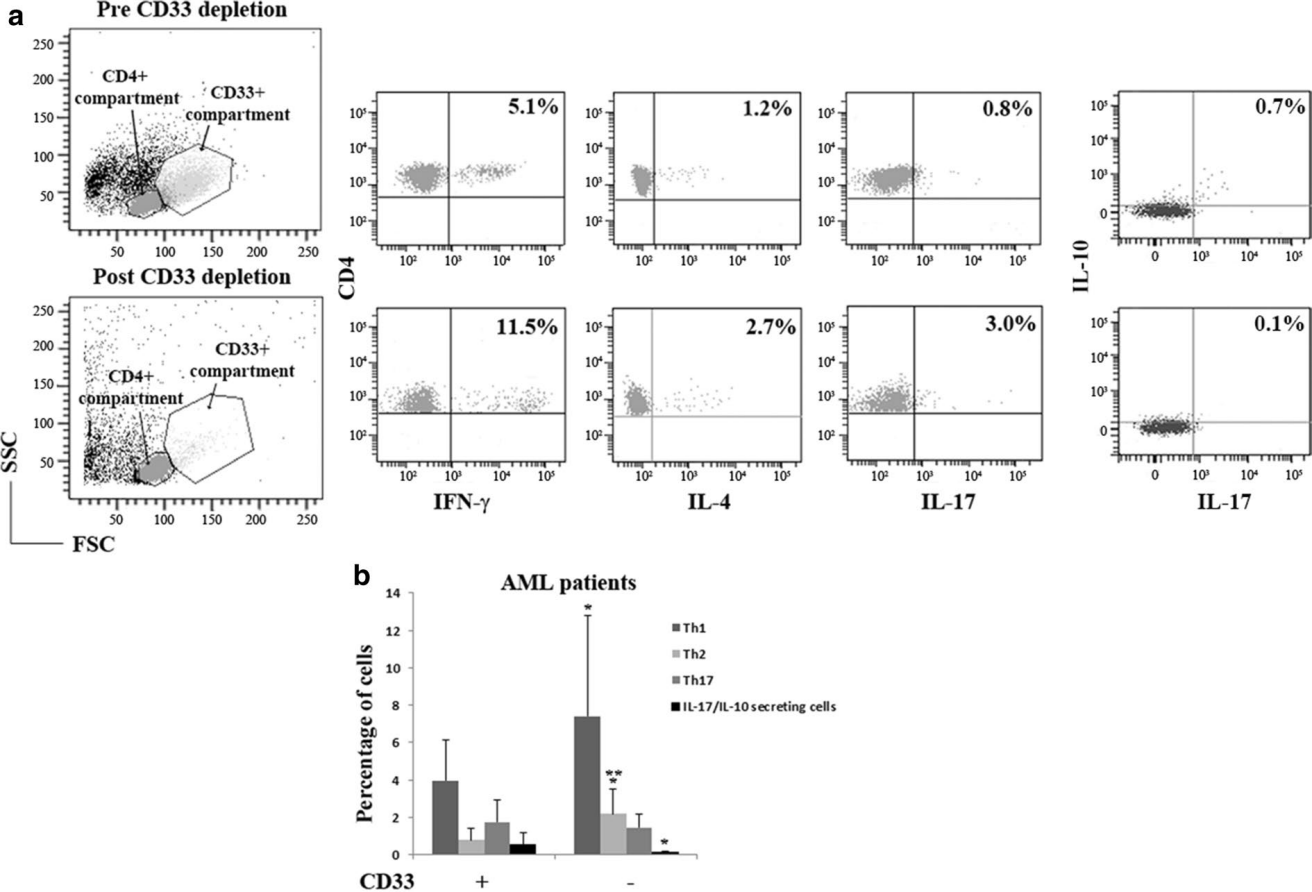
CD33+ cells are required for maintaining the altered cytokine pattern produced by T cells. **a** The scatter plot shows the blast compartment before and after the CD33 depletion. The dot plots show how the intracellular cytokine production changes before and after blast depletion on the same patient. All samples were stimulated with IL-6 for 24 h and PMA + I for 5 h. **b** Pooled data are shown as mean and standard deviation (*P < 0.05, ***P < 0.0005).

To provide other evidence of the blast action on CD4+ cells, we characterize the pattern of cytokine expression in AMLs before and after depletion of CD33+ blasts (Figure 5a). When patient CD4+ T cells were depleted of CD33+ cells, the former regained a capacity to produce IFN-γ and IL-4 similar to that of HV (*P* = 0.007 and *P* = 0.0001, respectively) whereas Th17 cell frequency tended to decrease but not significantly probably due to the high standard deviation among samples (Figure 5b). Interestingly, IL-17/IL-10-releasing cells significantly decreased after CD33+ removal, suggesting the involvement of blasts also in maintaining the immunosuppressive state in AML patients.

## Discussion

AML patients at the onset of disease and during chemotherapy are at high risk of severe and potentially fatal infections [2–4], but such conditions cannot be attributed to neutropenia alone. Indeed, concomitant reduced immune surveillance [9, 10, 12, 13], favors this infectious trend, worsens prognosis and limits therapeutic possibilities [3]. Multiple mechanisms of immunosuppression have been identified including indoleamine 2, 3-dioxygenase [9] and CD200 glycoprotein overexpression [10], an enhanced Treg activity [11], an impaired dendritic cell maturation [12] and PD1- PDL1 axis alteration [13].

More recently, another immunosuppressive mechanism was described, attributing defective immunological synapse formation to T cells [6]. All these mechanisms cooperate to suppress immune control on leukemia cells and infections, and also reduce the effect of vaccination or other adoptive T cell transfer strategies [7]. Nevertheless, the immune system may also be effective in controlling AML, as occurs in hematopoietic stem cell transplantation [8], and exploring additional ways to use such weapon could have a strong impact on the prognosis of these patients.

To our knowledge no convincing correlation has yet been found between immunosuppression, or specific T cell dysfunction, in AML and the infections to which AML patients are susceptible. Th17 cells, a particular subset of CD4+ cells and their respective cytokines, play a pivotal role in the inflammatory response and autoimmune diseases [15–18] and also direct the defense against bacterial and fungal infections of the gastrointestinal tract, skin, airways and lungs [26–28]. However, their function in several tumor types is controversial [19, 21, 22], and their involvement in hematological malignancies, in particular AML, remains to be defined [29–36]. Although our results confirmed previously published data [31, 33–36] showing statistically higher Th17 cell percentages in the peripheral blood of newly diagnosed AML patients compared to HV, the alteration in our study was observed together with a significant reduction in Th1 and Th2 frequencies. Moreover, a substantial increase in Tregs was observed, as previously reported by Szczepanski et al. [11].

Several studies hypothesized a role for Th17 cells in the pathogenesis of AML, but conflicting data on the different prognostic significance assigned to these cells [31–33] suggest an incomplete understanding of the mechanisms involved. In our opinion, the clinical presentation of AML patients, frequently affected by severe fungal or bacterial infections, is the most important event to be taken into account together with the increased percentage of Th17 cells [31, 33, 36], as confirmed in our experiments. Indeed, these two events are clearly conflicting, given the physiological role of defense of Th17 cells. For this reason, we also performed a more in-depth investigation into the ability of Th17 cells to produce IL-17 simultaneously with other cytokines, focusing our research on IL-10 [23] and IFN-γ [21]. A simultaneous production of these cytokines has already been demonstrated in Th17 [21–23, 25, 44], in line with their plasticity [14], and may also be a sign of their epigenetic transdifferentiation into other T cell types, such as Tregs [22, 23], which, themselves, may differentiate into FOXP3+ IL-17A cells [44] or Th17 Th1-like cells, secreting IFN-γ [20, 21].

We observed a significantly higher increase in the frequency of CD4+ IL-17A+/IL-10+ secreting cells in AML patients than in HV, whereas the percentage of IL-17A+/IFN-γ+ cells remained unchanged. Our results thus suggest that the substantial imbalance between IL-17/IL-10-producing cells (the involvement of FoxP3+IL-17A+IL-10+ cells cannot be excluded) and IL-17A/IFN-γ-producing cells, together with a reduced frequency in Th1 and Th2 cells, may act as an additional immunosuppressive factor in these patients, altering the physiological role of Th17, contributing to the infections and probably promoting leukemia escape. Furthermore, as Th17 cells are long-lived cells with a stem-like molecular signature [37], their immunosuppressive capacity in leukemia may be powerful and more durable.

Moreover, we demonstrated that the immune response of CD4+ cells isolated from patients was strongly reduced against an infective antigen of fungal origin and, notably, that the selective depletion of Th17 cells from the culture, led to a restoration of IFN-γ production.

To investigate the role of circulating leukemic blasts in the observed alteration, we selected CD33+ cells after depletion of myeloid differentiated cells. All the changes observed in Th17 were induced in vitro by CD33+ leukemic cells, as confirmed by direct and indirect co-cultures of healthy CD4+ cells and AML peripheral blasts. Given that in both co-culture the intensity of T alterations were similar, we hypothesized that leukemic cells action was mediated by soluble factors.

Moreover, patient T cells, depleted of CD33+ blasts, regained the capacity to produce levels of IFN- γ and IL-4 similar to those of HV and showed a decreased ability to simultaneously produce IL-17 and IL-10. Therefore all of the above data suggested the involvement of blasts also in maintaining the immunosuppressive state in AML patients.

## Conclusions

Aside from the known immunosuppressive mechanisms of AML [9–13], we identified a novel process induced by leukemic cells via soluble factors through which Th17 are converted into IL-17/IL-10-secreting cells, creating an environment with reduced immune control and thus probably favoring leukemia immune escape. Moreover, for the first time we can hypothesize a direct connection between the severe infective problems of AML patients and specific T cell alterations. The frequency of the immunosuppressive IL-17/IL-10-secreting cells in patients, needs more investigations but could represent a novel, simple, prognostic tool to identify the relative risk of severe infections and, perhaps, an increased risk of relapse for AML. Finally, although the role of Th17 cells in AML warrants further investigation, their long-lasting activity and plasticity could be used to convert these cells towards a tumor suppressor activity, making them a potential target for AML immunotherapy.

## Abbreviations

AML: acute myeloid leukemia
Th17: IL-17 T helper
Treg: T regulatory
*C. Albicans*: *Candida Albicans*
HV: healthy volunteers
FAB: French-American-British
PBMCs: peripheral blood mononuclear cells
FBS: fetal bovine serum
PMA: phorbol 12-myristate-13-acetate
BSA: bovine serum albumin
CMAC: 7-amino-4-chloromethylcoumarin
FSC: forward scatter
SSC: side scatter
